# Exploring the potential targets of the *Abrus cantoniensis* Hance in the treatment of hepatitis E based on network pharmacology

**DOI:** 10.3389/fvets.2023.1155677

**Published:** 2023-03-23

**Authors:** Ziheng Xu, Can Wang, Zuxiang Luan, Dapei Zhang, Baiqing Dong

**Affiliations:** ^1^School of Public Health and Management, Guang University of Chinese Medical, Nanning, Guangxi, China; ^2^Guangxi Key Laboratory of Veterinary Biotechology, Guangxi Veterinary Research Institute, Nanning, Guangxi, China; ^3^Department of Employment, Nanning Normal University, Nanning, Guangxi, China

**Keywords:** *Abrus cantoniensis* Hance, hepatitis E, network pharmacology, PI3K-Akt signaling pathway, β-sitosterol, Soyasapogenol E, Stigmasterol, Enoxolone

## Abstract

Hepatitis E is a disease of public health significance caused by the cross-species transmission of zoonotic hepatitis E virus (HEV) infection. There are no specific drugs. In this study, network pharmacology was used to reveal the mechanism of treatment of the active constituents of the *Abrus cantoniensis* Hance on hepatitis E. Based on the previously published representative components of *A. cantoniensis* Hance, we were screened the active components with OB ≥ 20% and DL ≥ 0.1 in *A. cantoniensis* Hance based on the TCMSP, predicted the target online through Swiss target prediction, and integrated the hepatitis E target in the GeneCards and DisGenet databases. Then, the core target was screened and the GO and KEGG enrichment and the network of the drug-active-ingredient-disease-pathway-target analysis were performed by the Cytoscape software. There were 11,046 hepatitis E targets, including PI3K-AKt, SRC, MAPK, PTPN11, EGFR, STAT1 and so on. The core ingredients include Oleanolic acid, Butin, β-sitosterol, Soyasapogenol E, 5,7-dihydroxy-2-methyl-8-[(2S,3R,4S,5S,6R)-3,4,5-trihydroxy-6-(hydroxymethyl)oxan-2-yl]oxychromen-4-one, Stigmasterol, Emodin, Physcion, and Enoxolone. A total of 1,410 GO enrichment results of core targets, including 1,246 biological process, 51 cell composition and 113 molecular function results. KEGG pathway was enriched in 150 related pathways, suggesting that *A. cantoniensis* Hance acts on cancer signaling pathway, endocrine resistance pathway, PI3K-AKt signaling pathway, MAPK, TNF and other signaling pathway. Through key components such as Oleanolic acid, Butin, β-sitosterol, Stigmasterol, and Enoxolone and other components interferes with AKT1, IL-6 and TNF, and regulates pathway in cancer, PI3K-AKt signaling pathway and MAPK pathway to play a therapeutic role in hepatitis E.

## Introduction

Hepatitis is one of the major national and global health challenges (1). Hepatitis E caused by fecal-oral transmission of zoonotic hepatitis E virus (HEV) is one of the major public health hazards (2). Genotypes 1 and 2 HEV are restricted to humans and often associated with large outbreaks and epidemics in developing countries with poor sanitation conditions, whereas genotypes 3 and 4 HEV infect humans, pigs and other animal species and are responsible for sporadic cases of hepatitis E (2–4). HEV not only has a wide range of animal hosts, but also has the characteristics of the cross-species transmission (4, 5). According to data released by the World Health Organization (WHO), about 20 million people worldwide are infected with hepatitis E every year, more than 3 million acute cases of hepatitis E, and nearly 70,000 deaths related to hepatitis E. Especially in developing countries or regions, the prevalence of hepatitis E is high. But in recent years, with the expansion of the transmission range, HEV has also become increasingly serious in developed countries. It is considered to be a new global public health problem with serious threats, which can particularly cause significant mortality in pregnant women and patients with cirrhosis (6), and there is no effective treatment so far. Recently, it has been found that immunocompromised patients (such as organ transplant recipients) are more likely to develop chronic hepatitis E (7). In pregnant women and patients with underlying chronic liver disease, acute hepatitis virus infection can develop into severe hepatitis and liver failure, directly causing the mortality rate of pregnant women and underlying chronic liver disease patients as high as 25 and 75%, yet there is no effective treatment. Currently, the Chinese-developed HEV vaccine, HEV-239, has been used in China for many years (8), but has not been licensed for use outside China. Drug therapy is the most widely used method for hepatitis E treatment at present. Although there are reports that some drugs such as ribavirin, interferon-alpha and Qingkailing injection have some therapeutic effects, none of them are characteristic drugs of HEV (9). Moreover, ribavirin is not a panacea, and the administration of ribavirin for pregnant women is highly debatable. In order to significantly improve the effective rate of treatment, shorten the treatment time, reduce the incidence of adverse reactions, reduce the cost of treatment, and reduce the burden on patients, it is urgent to develop efficient, drug-resistant and specific hepatitis E treatment drugs to help realize the goal of eliminating viral hepatitis as a major public health threat by 2030 proposed by the WHO (10).

*Abrus cantoniensis* Hance is the whole dried plant of leguminous plant *A. cantoniensis* Hance, which is a genuine regional drug in Guangxi, China. It tastes sweet and slightly bitter, and has the effects of dampness and get rid of jaundice, clearing heat and detoxifying, soothing liver and relieving pain. It is used for dampness-heat jaundice, side and rib discomfort, abdominal distension pain, and mammarectomy pain (11). It is often used in the treatment of chronic hepatitis B and other liver diseases. In the treatment of chronic hepatitis B, compound composed of chicken bone herb as the main drug combined with Entecavir can not only enhance the inhibition of HBV DNA replication, but also improve liver function indicators and delay the progression of liver fibrosis (10). In particular, through key components such as abrine, abrisapogenol, soyasapogenol and other components regulates hepatitis B signaling pathway to play a therapeutic role in hepatitis B (10). In addition, there have been studies on the combined treatment of 50 cases of toxic hepatitis in 8 weeks with traditional Chinese medicines such as *A. cantoniensis* Hance, Yinchen and gardenia, and the total effective rate is high (9, 10). So that *A. cantoniensis* Hance has significant effects on liver protection, anti-inflammatory, antiviral and immune regulation (10). However, its therapeutic effect on hepatitis E and its inhibitory effect on HEV have not been reported.

Network pharmacology is widely used in the field of drug research and development. Based on the core idea of “multi-component, multi-target and multi-pathway” of systems biology, it selects potential targets and pathways by calculating the network parameters of drug component targets, interprets the potential biological mechanism of Traditional Chinese medicine treatment of diseases, and discovers the basis of potential pharmacodynamic substances (12). In this study, network pharmacology was used to explore the mechanism of action of *A. cantoniensis* Hance in the treatment of hepatitis E, which provided theoretical support for subsequent drug development and mechanism verification.

## Materials and methods

### Predict active ingredients of *A. cantoniensis* Hance

The effective ingredients were obtained using “*A. cantoniensis*” as the keyword, searching through Traditional Chinese Medicine Systematic Pharmacology technology platform (TCMSP, https://tcmspw.com/tcmsp.php) (12), oral bioavailability (OB) ≥ 20%, and drug-likeness (DL) ≥ 0.1. Also, the main effective active ingredients of *A. cantoniensis* Hance were screened during this process.

### Acquire and collect action targets of *A. cantonensis* Hance

Based on previously published references (12), the TCMSP database and Swiss Target Prediction database were used to predict the target of related active ingredients first, and the target of active ingredients of *Abrus cantonensis* Hance was obtained afterward by importing it into the Uniprot online protein database (https://www.uniprot.org/).

### Acquire hepatitis E-related targets and map with *A. cantoniensis* Hance prediction targets

The hepatitis E-related targets were obtained using “hepatitis E” as a keyword and searched through the GeneCards database (https://www.genecards.org/) and OMIM database (https://omim.org/). Following, the duplicate target genes were deleted. Finally, both genes of epilepsy and *A. cantoniensis* Hance were mapped together to get a venny map.

### Protein–protein interaction network analysis and the core targets screen

The potential target of *A. cantonensis* Hance for hepatitis E treatment was introduced into the String database (https://string-db.org/), and the species “Homo sapiens” was selected to obtain a PPI network. The interaction score is set to ≥0.9 confidence level. The data was then imported into the Cytoscape 3.9.1 software.

### GO analysis and KEGG pathway enrichment analysis

A total of 62 core gene targets were imported into the Metascape database (https://metascape.org/gp/index) for Gene Ontology (GO) analysis and Kyoto Genomics and Genomics Encyclopedia (KEGG) analysis. Then, import KEGG path relationship file into the Cytoscape 3.9.1 software to predict the degree value, which was used to adjust the node size afterward. Finally, the “target -KEGG path” relationship network diagram was generated.

### Construct and analyze “drugs-active ingredients-diseases-pathway-targets” network

The obtained potential targets, KEGG pathways, diseases, and *A. cantoniensis* Hance for hepatitis E treatment were sent to the Cytoscape software to construct a “drug-active ingredients-diseases-pathway-targets” regulatory network diagram. Then, analyse the potential relationship between genes and components of *A. cantoniensis* Hance by a network diagram.

## Results

### Screen active ingredients of *A. cantoniensis*

We set OB ≥ 20% and DL ≥ 0.1 to obtain 9 main active ingredients of *A. cantoniensis* Hance, including Oleanolic acid, Butin, β-sitosterol, Soyasapogenol E, 5,7-dihydroxy-2-methyl-8-[(2S,3R,4S,5S,6R)-3,4,5-trihydroxy-6-(hydroxymethyl)oxan-2-yl] oxychromen-4-one, Stigmasterol, Emodin, Physcion, and Enoxolone (Supplementary Table S1), based on the TCMSP database.

### Predict potential targets of *A. cantoniensis* Hance components in hepatitis E

After repeated targets were removed, 285 targets of *A. cantoniensis* Hance active ingredients were obtained based on the Swiss target prediction (Supplementary Figure S1).

All 11,046 hepatitis E targets were obtained based on the GeneCards database and PharmGKB database platform. Intersecting the target related to *A. cantoniensis* Hance with the disease target, we obtained the Wayne diagram of *A. cantoniensis* Hance target for hepatitis E treatment (Supplementary Figure S1). 258 common targets were obtained, and the three effective components of *A. cantoniensis* Hance were mapped with hepatitis E genes (Supplementary Figure S1). It was found that there were multiple common targets of 9 components and hepatitis E and could play its role through multiple targets (Supplementary Figure S2).

### Screening of core targets

**Figure 1:**
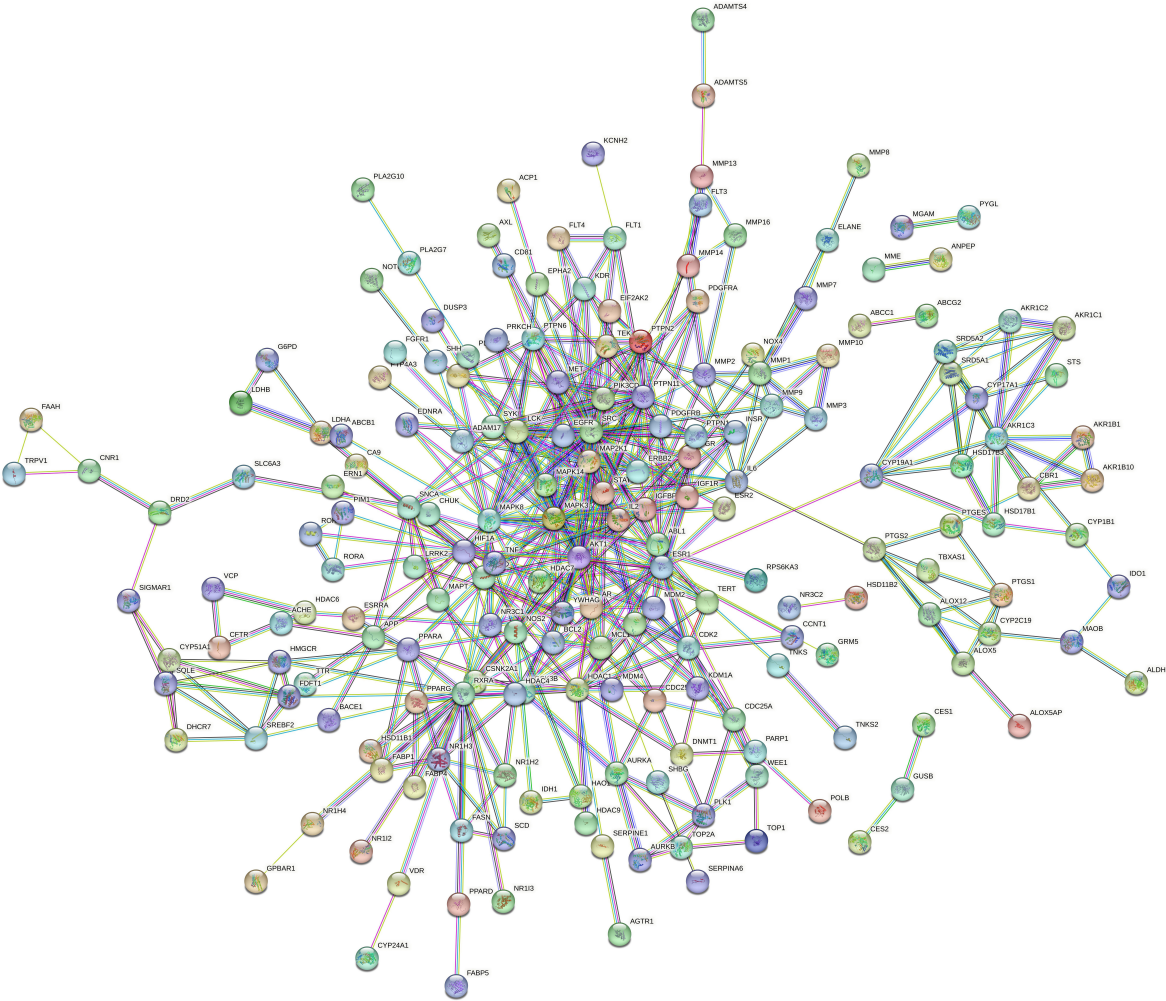
PPI of interacting target.

As shown in Figure 1, the PPI network consists of 258 nodes and 501 edges.

**Figure 2:**
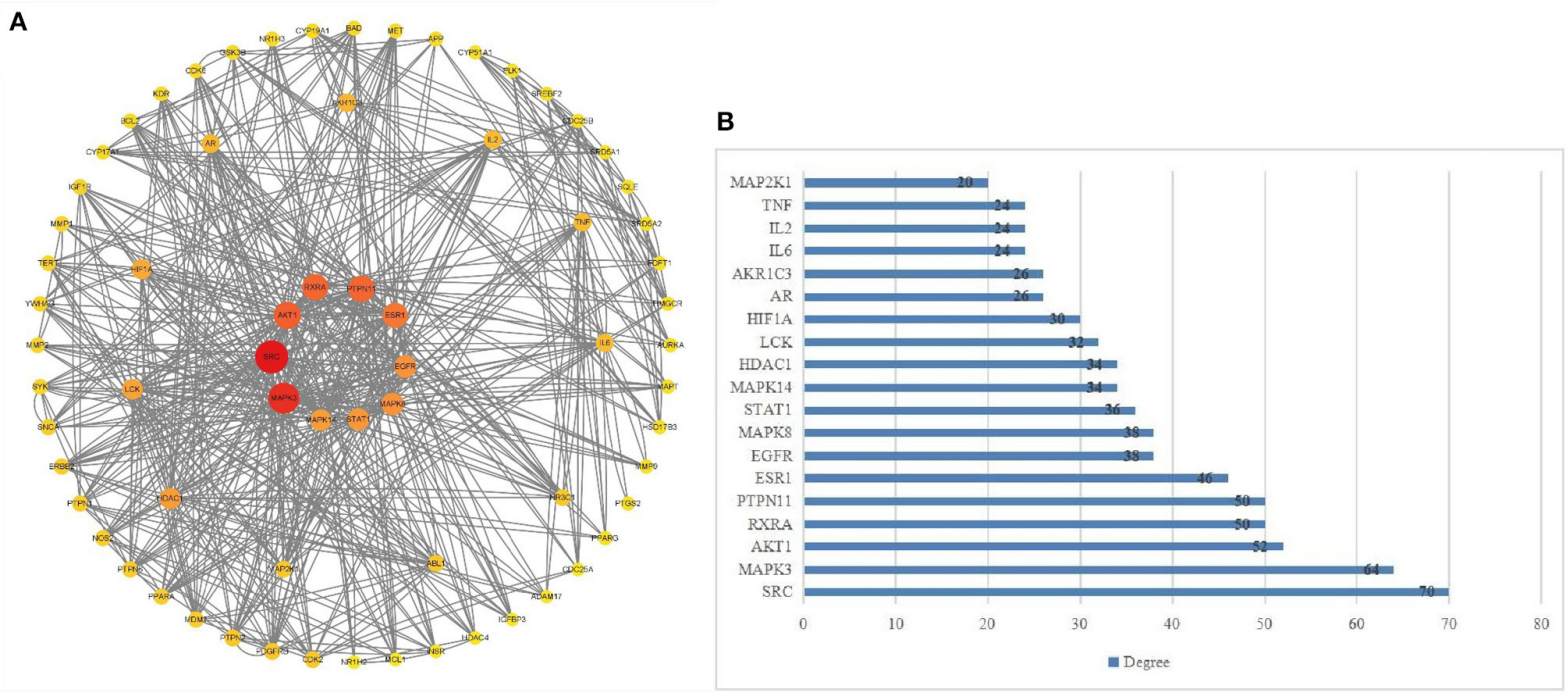
**(A, B)** Core target network diagram. The target near the center of the circle is the core target.

Based on the results of PPI, Cytoscape 3.9.1 software was utilized to sequence and visualize the intersection targets according to degree (maximum degree of inner circle), and to draw bar graph/target network graph. We obtained 62 core gene targets (Figure 2A). The top 10 targets with the highest correlation were SRC (70), MAPK3 (64), AKT1 (52), RXRA (50), PTPN11 (50), ESR1 (46), EGFR (38), MAPK8 (38), STAT1 (36), and MAPK14 (34) (Figure 2B). These results suggest that these genes may be potential targets for the treatment of hepatitis E.

### Biological function and pathway analysis

**Figure 3:**
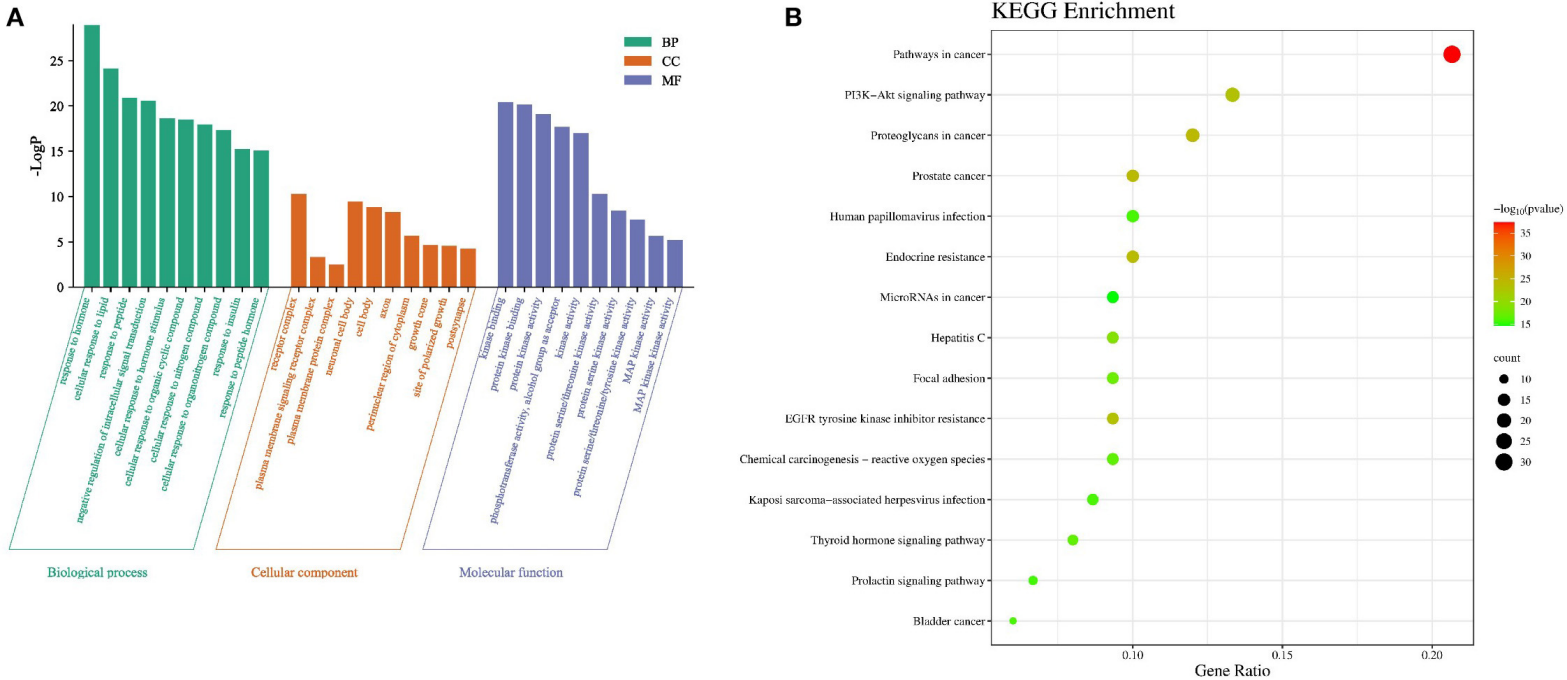
**(A, B)** GO and KEGG enrichment analysis results.

There were 1,410 GO enrichment results of core targets, including 1,246 biological process (BP), 51 cell composition (CC) and 113 molecular function (MF) results (Figure 3A). The results of GO annotation showed that the biological functions of *A. cantoniensis* Hance, the key target of hepatitis E treatment, were mainly involved in response to hormone, negative regulation of intracellular signal transduction, neuronal cell body, cell body, protein kinase activity, and other biological processes responsiveness. It showed that the effective components of *A. cantoniensis* Hance played a role in treating hepatitis E by regulating various biological pathways.

There were 150 KEGG enrichment-related pathways (Figure 3B). The enrichment results of KEGG pathway show that the key target genes of *A. cantoniensis* Hance in treating hepatitis E were significantly enriched in the several signal pathways, including pathways in cancer, PI3K-AKt signaling pathway, EGFR tyrosine kinase inhibitor resistance, Hepatitis C pathway, Thyroid hormone signaling pathway, and etc., besides, the MAPK signaling pathway, p53 signaling pathway, B cell receptor signaling pathway, Hepatitis B, T cell receptor signaling pathway, TNF signaling pathway and so on, were also included (*p* < 0.05). It is speculated that these pathways may also be in *Abrus cantonensis* Hance.

### Construct a drug-active ingredient-disease-pathway-target network

**Figure 4:**
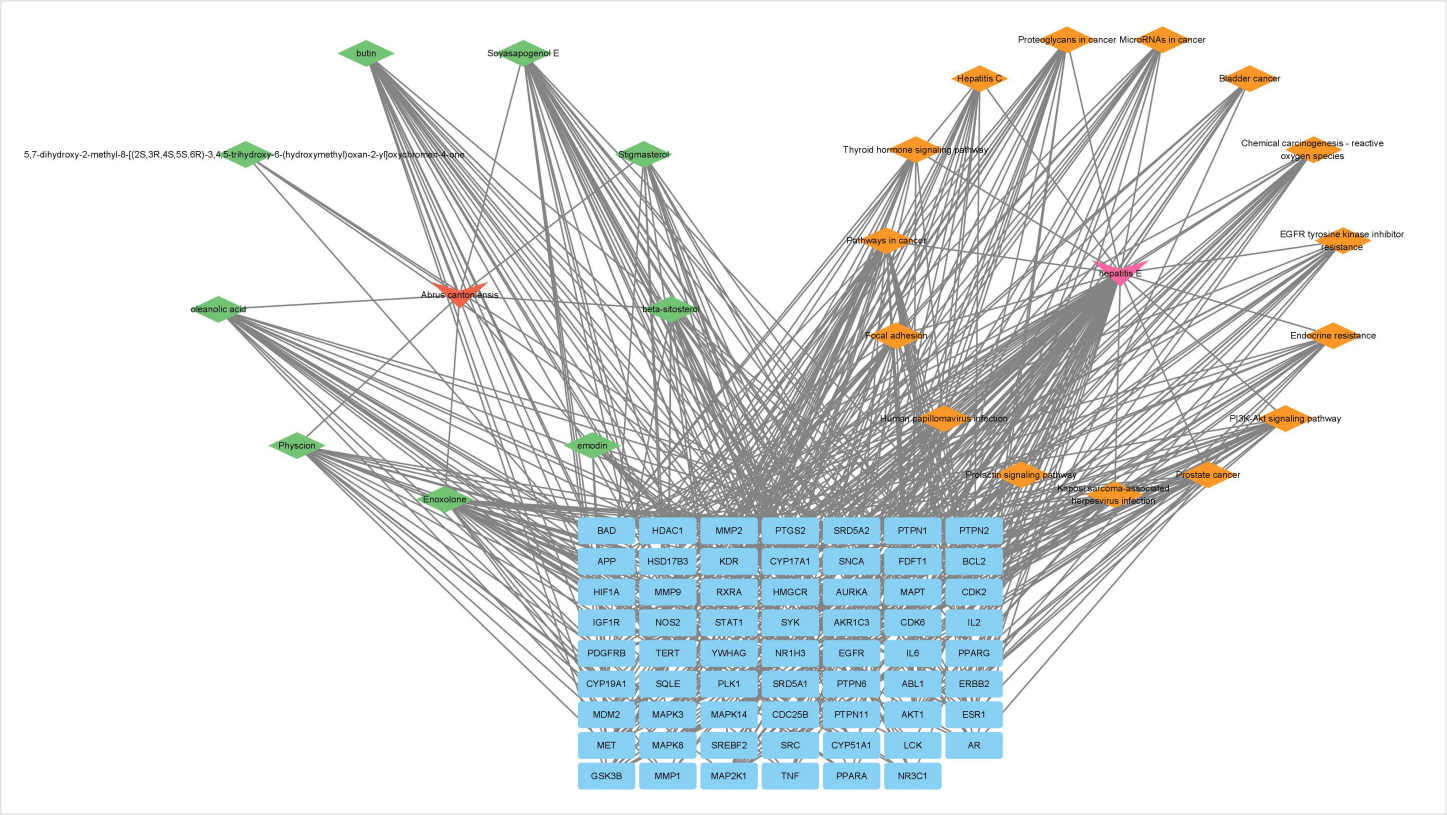
Drug-active ingredient-disease-pathway-target network.

Visualization of KEGG concentration analysis correlated with hepatitis E development, 9 key active components acting 15 key pathways associated with hepatitis E, including PI3K-AKt signaling pathway, MAPK signaling pathway, IL-2/IL-6 signaling pathway and TNF signaling pathway (Figure 4). It is indicated that *Abrus cantonensis* Hance exerts potential therapeutic effect for hepatitis E through “multiple component, multitarget and multiple pathway.”

## Discussions

The results of this study showed that the active constituents of *A. cantoniensis* Hance acted on multiple targets and pathways. Among them, Oleanolic acid, Butin, 5,7-dihydroxy-2-methyl-8-[(2S,3R,4S,5S,6R)-3,4,5-trihydroxy-6-(hydroxymethyl)oxan-2-yl]oxychromen-4-one, β-sitosterol, Soyasapogenol E, Stigmasterol, Emodin, Physcion, and Enoxolone are the core chemical components in the treatment of hepatitis E by *A. cantoniensis* Hance. It has been shown that hepatitis E can be treated by compound glycyrrhizin (SNMC), which is a compound consisting of enoxolone and glucuronic acid (13). In addition, glycyrrhizic acid can inhibit the inflammatory response of liver, reduce the pathological injury of liver, and restore the normal function of damaged liver cells (14). Therefore, this study is the first to confirm that enoxolone, the active ingredient of *A. cantoniensis* Hance, can play a role in the treatment of hepatitis E. In addition, Butin, β-sitosterol and stigmasterol have been shown to have therapeutic effects on Hepatitis B (10). This study also confirmed that it has the same therapeutic effect on hepatitis E, possibly because these ingredients can inhibit the inflammatory response and play the same role. In addition, 5,7-dihydroxy-2-methyl-8-[(2S,3R,4S,5S,6R)-3,4,5-trihydroxy-6-(hydroxymethyl)oxan-2-yl]oxychromen-4-one, Soyasapogenol E, Emodin and Physcion were the first reported active ingredient of *A. cantoniensis* Hance in the treatment of hepatitis E.

The target organ of HEV is the liver, so at the beginning of infection, Alanine aminotransferase (ALT) and Aspartate aminotransferase (AST) in the liver are significantly elevated. Suggesting that these enzymes play a role in liver damage. After the action of *A. cantoniensis* Hance, the binding force of some protein kinases and the activity of some protein kinases are also enhanced. It may be that the effective ingredients of *A. cantoniensis* Hance regulate the enzyme activity in the body, so as to play the role of liver protection. In addition, nervous system complication is the most common symptom of HEV infection, and also the most concerned point. Shi et al. (15) found that HEV can infect and replicate in the brain and spinal cord tissues of gerbils, and the expression of tight junction protein Zonula occlidens-1 is decreased in the infected gerbils, while the number of astrocytes is significantly increased. This result confirms that HEV can destroy the blood brain barrier and is directly related to the damage of the nervous system. In the GO enrichment study, neuronal cell body was found to be highly enriched in this study, indicating that the active components of *A. cantoniensis* Hance can effectively improve neuronal cell body, so as to effectively improve the neurological complications caused by HEV.

The results of this study show that The active components of the *A. cantoniensis* Hance can affect cancer signaling pathway, endocrine resistance, PI3K-AKt signaling pathway, MAPK signaling pathway, IL-2/IL-6/IL-17 signaling pathway and TNF signaling pathway. These signaling pathways play an important role in the occurrence and development of hepatitis and liver cancer. Studies have shown that IL-6 can be expressed in hepatocytes, and the main activators of IL-6 expression are IL-1b and tumor necrosis factor (TNF-α) (16). Activation of the Phosphoinositol 3 kinase (PI3K)-protein kinase activates the B(PKB)/Akt pathway, in which JAK phosphorylates and activates PI3K and then phosphorylates some phosphatidylinositol to phosphatidylinositol-4, 5-diphosphate (PIP2) and phosphatidylinositol-3,4,5-triphosphate (PIP3). PIP3, in turn, phosphorylates and activates PKB/Akt, which is recruited into the plasma membrane. This PI3K/Akt pathway contributes to the activation of NF-κB (16). In this study, IL-6 is the key gene in the treatment of hepatitis E, and the regulation of PI3K-Akt signaling pathway is the potential mechanism of the treatment of hepatitis E by the *A. cantoniensis* Hance. Tumor necrosis factor (TNF) signaling pathway is a classic inflammatory signaling pathway, which can promote the generation of many diseases, including liver diseases. TNF signaling pathway can be regulated upstream and downstream with other inflammatory proteins and cytokines, and then fluctuate other inflammatory pathways or regulate each other, jointly promoting the development of diseases. Recent evidence suggests that cathepsin C interacts with TNF-α/p38 mitogen activated protein kinase (MAPK) signaling pathways to promote hepatocellular carcinoma proliferation and metastasis (17). TNF and IL-17 have the potential to promote liver inflammation and fibrosis induced by activation of Nod-like receptor heat protein domain associated Protein 3 (NLRP3) inflammasome (18). NLRP3 is also considered as a potential pathogenic mechanism involved in HEV infection (19). Therefore, the active constituents of *A. cantoniensis* Hance can also play a role in the treatment of hepatitis E by regulating TNF signaling pathway. In addition, the MAPK signaling pathway can also mediate the development of liver diseases (20), and the active ingredients of the *A. cantoniensis* Hance can regulate the above inflammatory signaling pathway, thus playing a potential therapeutic role.

In conclusion, this study reveals the potential mechanism of *A. cantoniensis* Hance in the treatment of hepatitis E through network pharmacology. We found that *A. cantoniensis* Hance may enhance the activity of protein kinases, promote the growth of neuronal cells body, and regulate hepatitis related signaling pathways (PI3K-AKt, MAPK, IL-2/IL-6/IL-17 signaling pathway and so on), which can serve as a reference in hepatitis E treatment. However, network pharmacology analysis only provides a theoretical prediction, which needs to be verified by further experiments.

## Data availability statement

The original contributions presented in the study are included in the article/Supplementary material, further inquiries can be directed to the corresponding author.

## Author contributions

ZX, CW, and ZL completed data analysis and the draft and contributed to the manuscript. DZ assisted in this manuscript. BD provided the funding of research, reviewed, and approved the final manuscript. All authors contributed to the article and approved the submitted version.

## Supplementary material

Supplementary Table S1 Chemical composition of *A. cantoniensis* Hance.

Supplementary Figure S1 Intersection target of *A. cantoniensis* Hance and hepatitis E.

Supplementary Figure S2 Map of core target.
