# Supplementary material for: Exploring the potential targets of the Abrus cantoniensis Hance in the treatment of hepatitis E based on network pharmacology
Source: Front Vet Sci. 2023 Mar 23;10:1155677. doi: 10.3389/fvets.2023.1155677 (PMC10076809; doi:10.3389/fvets.2023.1155677)
Supplement: Supplementary Table S1 — Chemical composition of A. cantoniensis Hance. [file Table_1.docx]

**Table S1** Chemical composition of *Abrus cantoniensis* Hance

| No. | Molecule Name | Molecular Weight | OB (%) | [DL](https://old.tcmsp-e.com/tcmspsearch.php?qr=Abri Herba&qsr=herb_en_name&token=8a474b210f1a32f558c3fffd60e12c69) | Molecular ID |
| --- | --- | --- | --- | --- | --- |
| 1 | [5,7-dihydroxy-2-methyl-8-[(2S,3R,4S,5S,6R)-3,4,5-trihydroxy-6-(hydroxymethyl)oxan-2-yl]oxychromen-4-one](https://old.tcmsp-e.com/molecule.php?qn=13323) | 370.34 | 25.82 | 0.42 | MOL013323 |
| 2 | [Oleanolic acid](https://old.tcmsp-e.com/molecule.php?qn=263) | 456.78 | 29.02 | 0.76 | MOL000263 |
| 3 | [Butin](https://old.tcmsp-e.com/molecule.php?qn=2975) | 272.27 | 69.94 | 0.21 | MOL002975 |
| 4 | [β-sitosterol](https://old.tcmsp-e.com/molecule.php?qn=358) | 414.79 | 36.91 | 0.75 | MOL000358 |
| 5 | [(4aR,6aR,6aS,6bR,8aR,9S,10S,12aR,14bS)-10-hydroxy-2,2,4a,6a,6b,9,12a-heptamethyl-9-methylol-3,5,6,6a,7,8,8a,10,11,12,13,14b-dodecahydro-1H-picen-4-one](https://old.tcmsp-e.com/molecule.php?qn=3651) | 456.78 | 37.64 | 0.75 | MOL003651 |
| 6 | [Stigmasterol](https://old.tcmsp-e.com/molecule.php?qn=449) | 412.77 | 43.83 | 0.76 | MOL000449 |
| 7 | [Emodin](https://old.tcmsp-e.com/molecule.php?qn=472) | 270.25 | 24.4 | 0.24 | MOL000472 |
| 8 | [Physcion](https://old.tcmsp-e.com/molecule.php?qn=476) | 284.28 | 22.29 | 0.27 | MOL000476 |
| 9 | [18β-glycyrrhetinic acid](https://old.tcmsp-e.com/molecule.php?qn=4804) | 470.76 | 22.05 | 0.74 | MOL004804 |
