# Supplementary material for: Exploring the potential targets of the Abrus cantoniensis Hance in the treatment of hepatitis E based on network pharmacology
Source: Front Vet Sci. 2023 Mar 23;10:1155677. doi: 10.3389/fvets.2023.1155677 (PMC10076809; doi:10.3389/fvets.2023.1155677)
Supplement: Supplementary Figure S1 — Intersection target of A. cantoniensis Hance and hepatitis E. [file Data_Sheet_1.docx]

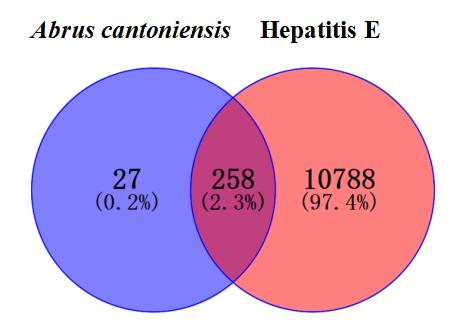


**FIGURE S1** Intersection target of *Abrus cantoniensis* Hance and Hepatitis E.


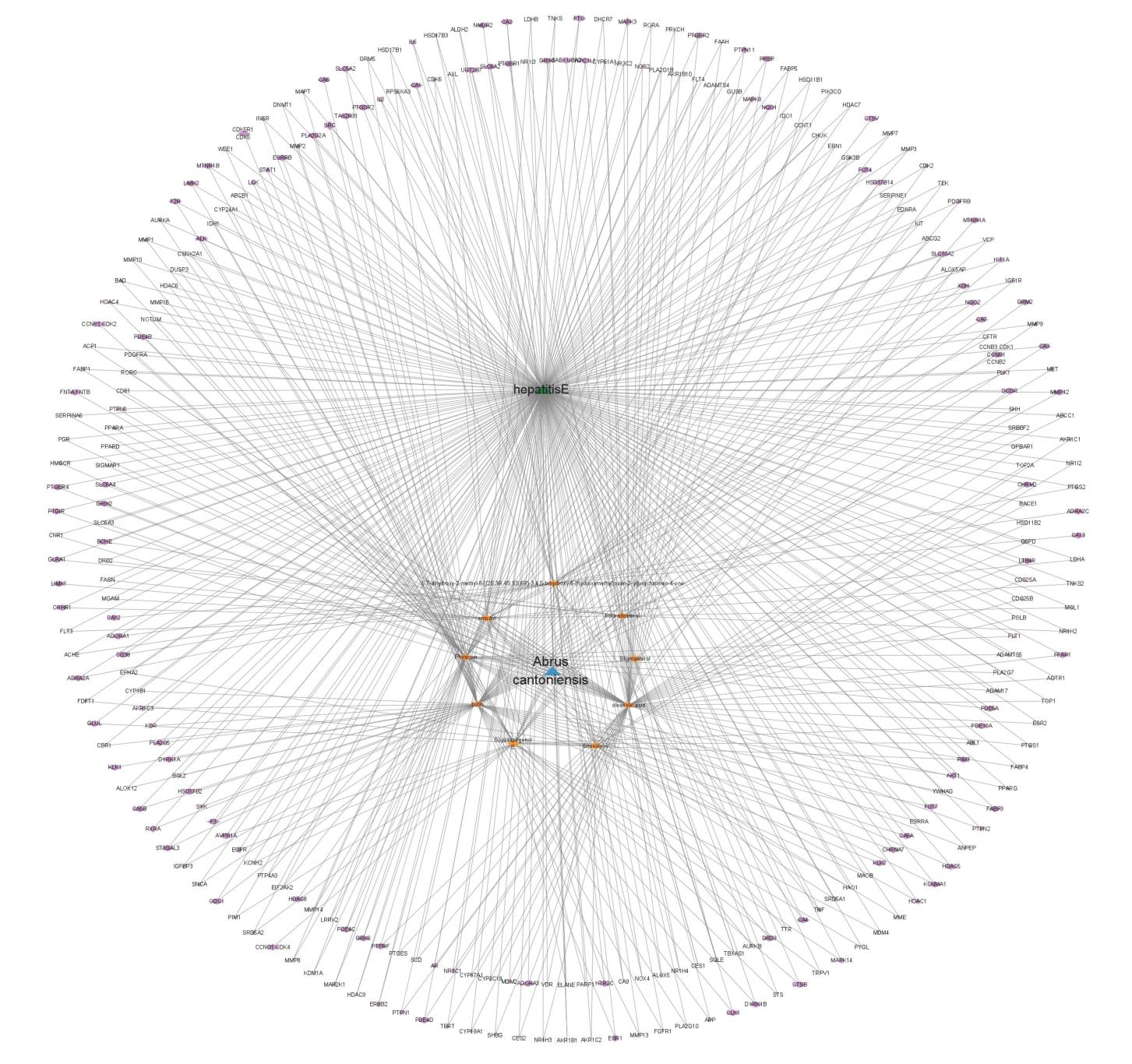


**FIGURE S2** Map of core target.
